# A Piece of the Puzzle: The Bone Health Index of the BoneXpert Software Reflects Cortical Bone Mineral Density in Pediatric and Adolescent Patients

**DOI:** 10.1371/journal.pone.0151936

**Published:** 2016-03-25

**Authors:** Michael M. Schündeln, Laura Marschke, Jens J. Bauer, Pia K. Hauffa, Bernd Schweiger, Dagmar Führer-Sakel, Harald Lahner, Thorsten D. Poeppel, Cordula Kiewert, Berthold P. Hauffa, Corinna Grasemann

**Affiliations:** 1 Pediatric Hematology and Oncology, Kinderklinik III, Universitätsklinikum-Essen and the University of Duisburg-Essen, Essen, Germany; 2 Pediatric Endocrinology and Diabetology, Kinderklinik II, Universitätsklinikum-Essen and the University of Duisburg-Essen, Essen, Germany; 3 Institute of Diagnostic and Interventional Radiology and Neuroradiology, Universitätsklinikum-Essen and the University of Duisburg-Essen, Essen, Germany; 4 Department of Endocrinology and Metabolism, Medical Center, Universitätsklinikum-Essen and the University of Duisburg-Essen, Essen, Germany; 5 Clinik for Nuclear Medicine, Universitätsklinikum-Essen and the University of Duisburg-Essen, Essen, Germany; Leeds Beckett University, UNITED KINGDOM

## Abstract

**Introduction:**

Suspected osteopathology in chronically ill children often necessitates the assessment of bone mineral density. The most frequently used methods are dual-energy X-ray-absorption (DXA) and peripheral quantitative computed tomography (pQCT). The BoneXpert software provides an automated radiogrammatic method to assess skeletal age from digitalized X-rays of the left hand. Furthermore, the program calculates the Bone Health Index (BHI), a measure of cortical thickness and mineralization, which is obtained from indices of three metacarpal bones. In our study, we analyzed the manner in which BHI information provided by BoneXpert compares with DXA or pQCT measurements in youths.

**Study Design:**

The BHI was retrospectively obtained using digitalized X-rays of the left hand and compared with the results of 203 corresponding DXA readings (Lunar Prodigy, GE Healthcare) of the lumbar vertebrae and femur as well as 117 pQCT readings (XCT 900, Stratec) of the distal radius.

**Results:**

The BHI values showed a strong positive correlation with the DXA readings at each and all lumbar vertebrae (L1 –L4: r = 0.73; *P* < 0.0001). The age-adjusted Z-score of L1 –L4 and the height-adjusted score showed a positive correlation with the BHI-SDS (standard deviation score, r = 0.23; *P* < 0.002 and r = 0.27; *P* < 0.001, respectively). Total bone mineral density, as assessed via pQCT, also positively correlated with the BHI (r = 0.39; *P* < 0.0001), but the trabecular values displayed only a weak correlation.

**Conclusions:**

The BHI obtained using BoneXpert can be a useful parameter in the assessment of bone health in children in most cases. This technique provides observer-independent information on cortical thickness and mineralization based on X-ray imaging of the hands.

## Introduction

The assessment of bone health in pediatric patients is a challenging task for the treating physician. Besides clinical information pediatricians must obtain and interpret a variety of radiographic and biochemical surrogate parameters of skeletal health (as reviewed by the Committee on Nutrition of the American Academy of Pediatrics[1]). While vitamin D status and bone turnover are mainly reflected through biochemical markers, bone mineral density (BMD) can be assessed using different radiographic techniques: dual energy X-ray absorption (DXA) scans[2,3], peripheral quantitative computed tomography (pQCT)[2,4–6] and radiogrammetric methods based on cortical thickness in regular X-rays[7,8].

While DXA scans represent the most widely accepted and used technique to assess BMD in adults, this method presents limitations for the use in pediatrics[2]. For example, the growing skeleton necessitates a correction of the obtained BMD reading for the size of the bone (e.g., the vertebra) at which the BMD was assessed. This is partly achieved by the (automated) calculation of Z-scores, which provide gender-specific and age-adapted standard deviation score values (SDS) for the DXA readings at vertebrae L1-L4 in pediatric patients.

Similar with the T-scores, which are SDS values used in adults to rank a BMD of the spine in reference to healthy thirty-year-old persons, the pediatric Z-scores refer to a healthy age-appropriate cohort of children and adolescents with normal growth. In youths with short stature, the Z-score underestimates the BMD, whereas in children with tall stature it overestimates the BMD of the individual patient[3].

As children with suspected osteopathology are often also affected by chronic disease or endocrine disorders resulting in deviant growth, a further correction of the Z-score for the actual height of the patient is necessary[2,6,9,10].

BMD measurement of peripheral sites (arm, leg) in children using pQCT, in which the calculation reconstructs the three dimensional structure of a bone, provides meaningful data in pediatrics, although the technique is not available at all sites[4].

In contrast, radiographs of the left hand are routinely used in pediatrics to determine the bone age of an individual patient for a variety of clinical questions. The methods that are most commonly used for the assessment of bone age from hand radiographs were developed by Greulich and Pyle[11] and by Tanner and Whitehouse[12]. Both methods are susceptible to substantial intra-and inter-observer variability and require an experienced interpreter of skeletal age determination at the site[13].

To facilitate the assessment of bone age, a number of computerized programs have been developed. Recently, Thodberg and others have developed and described such a program, BoneXpert, to calculate bone age from a digital hand radiograph[14–16].

Additionally, the software calculates the pediatric Bone Health Index (BHI), which describes bone mass as a function of the cortical thickness of three metacarpals and the metacarpal width and length[17]. The program also provides SDS values for the BHI readout, which are automatically calculated based on a large cohort of Caucasian children[17,18].

This study aimed to assess the automated BHI measurement calculated by the BoneXpert software compared with DXA and pQCT readings. We reviewed BHI readings from patients for whom DXA or pQCT measurements had been performed. Furthermore, we reviewed the results of the BHI results in a cohort of patients for which a biochemical workup of bone metabolism had been performed.

## Materials and Methods

### Left hand radiographs and BHI

We retrospectively reviewed the BHI and BHI SDS data of digitalized left hand radiographs (DICOM format) of a cohort of pediatric patients. The children had been seen in the pediatric endocrine or pediatric oncology outpatient clinic between March 2004 and June 2013 and had undergone a DXA or pQCT scan performed within an 8-month window relative to the hand x-ray. Patients from the following diagnostic groups were seen for follow-up visits: aberrant growth or pubertal development, other suspected endocrine disease, chronic hematologic conditions and survivors of pediatric malignancies (survivorship clinic). The radiographs of the left hand of each patient were carried out to obtain a skeletal age. Information concerning body weight, height and pubertal stages at the time of the X-ray were retrieved from the patient charts.

A total of 346 radiographs were submitted for BoneXpert-based analysis. The program was unable to analyze the hand radiographs of nine subjects (2.3%) due to a variety of issues as follows: image too sharp (1), bone age is too low or bones have abnormal shapes or incorrect hand pose/poor image quality (3), cortex inconsistencies (1), inconsistent lengths (3), or unable to determine bone age (1).

The remaining 337 images were analyzed using the software. The BHI and BHI SDS results were compared with the results of DXA- (n = 203) or pQCT scans (n = 117). For 23 BHI readings, both a DXA and pQCT scan were available within the 8-month window. A comprehensive biochemical assessment of bone metabolism corresponding to a hand X-ray was available in 114 cases. Forty of those BHI readings were compared only with the biochemical results, whereas for 74 readings, a corresponding DXA or pQCT scan was also available.

The study was performed in accordance with the ethical principles of the Declaration of Helsinki and with the approval of the local research ethics committee ('Ethikkommission der Medizinischen Fakultät der Universität Duisburg-Essen'; 15-6406-BO). A written informed consent was not obtained since the data were analysed anonymously. For detailed patient characteristics, refer to Table 1.

**Table 1:** Descriptive statistics of relevant patient clinical data.

	All (n = 337)	Boys (n = 198)	Girls (n = 139)
**Age (y)**	13.58 ± 3.57 (3.64–21.14)	13.3 ± 3.79 (4.32–20.02)	13.97 ± 3.21 (3.64–21.14)
**Bone Age (y)**	12.75 ± 3.58 (2.42–19) 333	12.46 ± 3.78 (2.42–19) 198	13.18 ± 3.22 (3.17–18) 135
**Weight SDS**	-0.3 ± 1.64 (-5.32–4.11) 283	-0.36 ± 1.58 (-5.32–4.01) 171	-0.23 ± 1.72 (-4.65–4.12) 112
**Height SDS**	-0.87 ± 1.18 (-4.06–2.73) 285	-0.87 ± 1.15 (-3.97–2.32) 173	-0.87 ± 1.22 (-4.06–2.73) 112
**BMI SDS**	0.17 ± 1.58 (-5.65–4.62) 283	0.13 ± 1.5 (-5.65–4.62) 171	0.23 ± 1.7 (-4.44–3.84) 112
**PH SDS**	-0.38 ± 1.11 (-3.14–2.33) 186	-0.38 ± 1.09 (-3.14–2.33) 106	-0.39 ± 1.14 (-3.14–1.99) 80
**TVBR SDS**	-0.6 ± 1.36 (-4.0–2.08) 175	-0.75 ± 1.46 (-4.0–1.88) 97	-0.43 ± 1.22 (-3.35–2.08) 78

Mean ± SD and range (in parentheses) are displayed, followed by the number of patients examined for the following parameters: age (in years), bone age (in years) assessed using the method of Greulich and Pyle, weight SDS, height SDS, body mass index SDS (BMI SDS), pubic hair stage SDS (PH SDS) and testicular volume/breast development stage SDS (TVBR SDS).

### BHI

Conventional radiographs of the anterior-posterior view of the left hand were obtained for the assessment of bone age for clinical reasons. Digital images were stored in picture archiving and communication system (PACS) in DICOM format (digital imaging and communications in medicine).

Bone age, BHI and BHI SDS were calculated using BoneXpert Software (BoneXpert version 2, Visiana, Holte, Denmark) as previously described by Thodberg and colleagues[17]. The method has been validated on a variety of patient groups and ethnicities [14,16,19–21].

Calculation of the BHI is a radiogrammetric method (sometimes called Digital X-ray Radiogrammetry (DXR)) that uses the dimensions in a plain radiograph to calculate an index. A bone index is not a direct assessment of BMD. Instead, the index rather represents bone mineral density divided by a power of a length to render the index more representative of the bone health of the pediatric subjects, whose size is highly variable. Concerning assessment in adults, Barnet and Nordin[7] have proposed the metacarpal index. Using cortical thickness (T), length (L) and width (W) of the three middle metacarpals, the MCI is computed as MCI = T/W, whereas the approximate expression of BHI is BHI = T/(LW)^0.33^.

BHI SDS enables for comparison of the observed BHI with the BHI of healthy subjects of the same gender and bone age. Such reference curves have been determined from Dutch boys and girls, and these values are used in BoneXpert to compute the BHI SDS values. This calculation is performed automatically based on the bone age derived from the same X-ray using BoneXpert.

### DXA

BMD was examined via DXA scanner, Lunar Prodigy, GE-Healthcare, Madison, WI, USA). Areal BMD (g/m^2^) was assessed at the lumbar spine (L1–L4; anteroposterior view) and the left femoral neck. Z-scores were calculated for the lumbar spine measurements based on the normative values for the corresponding age as provided by the manufacturer.[22–24]. A single investigator, blinded to the clinical status of the patients, was responsible for all BMD measurements.

Height adjusted Z-scores[25] (HAZ) of the BMD readings of the lumbar spine (L1–L4; anteroposterior view and of the femoral neck) were calculated based on the same reference data provided by the manufacturer after correction for “height age” (age at which the actual height at the time of the DXA reading corresponds to the 50th percentile for growth) based on normative data for a German population[26].

### pQCT

PQCT readings were obtained measuring the distal forearm of the non-leading hand using an XCT 900 system (Stratec Medizintechnik GmbH, Pforzheim, Germany). Of note, no normative data for pediatric ages is available for the XCT 900. Pediatric SDS values were calculated for total BMD and trabecular BMD using data from a cross calibration with an XCT 2000 device, as described by Rauch et al.[5,27].

### Laboratory testing

The biochemical tests included measurement of serum 25-OH vitamin D (ng/ml); 1,25-(OH)_2_ vitamin D (pg/ml); total serum alkaline phosphatase, TSAP (U/l); bone-specific alkaline phosphatase, BSAP (U/l) and plasma parathyroid hormone, PTH (pg/ml) levels. Additionally, the calcium to creatinine ratio (mg/mg) in urine and markers of bone resorption, including N-terminal telopeptide (NTX) (nmol bone collagen equivalent (BCE)/mmol creatinine) and deoxypyridinoline (DPD) (mg/g creatinine), were assessed in spot urine samples.

### Clinical parameters

Clinical parameters were obtained during regular visits in the outpatient clinic and were retrieved from the patient charts. Briefly, during each visit, a physical exam was performed assessing patient height, weight and pubertal staging according to Tanner stage.

Standing height was measured using a wall-mounted stadiometer (Ulmer Stadiometer, Busse Design, Elchingen, Germany) to the nearest mm. Weight was recorded to the nearest 0.1 kg using a digital scale (Seca, Hamburg, Germany). BMI was calculated from these data using the formula weight (kg)/(height² x m²). The measurements were transformed into SDS values based on a reference data set for German children[26]. An experienced pediatrician assessed the pubertal development according to the Tanner stages. Testicular volume was assessed using a Prader orchidometer. Pubertal status data were then transformed into SDS values based on the data reported by Mul et al.[28]. Conversion of pubertal stages into SDS was performed using the web application Puberty Plot S-plus package (http://vps.stefvanbuuren.nl/puberty; accessed 2016 February 16) designed by van Buuren and Ooms[29].

### Statistics

Values are expressed as the mean +/- standard deviation (SD) and range unless stated otherwise. Associations between single variables were assessed using the Spearman correlation coefficient. Statistical significance was assumed at *P <* 0.05. Linear regression, Spearman correlation analyses and Mann-Whitney tests were performed using PRISM 6 for MAC OS X (La Jolla, CA, USA).

The influence of height SDS, weight SDS, BMI SDS and puberty on BHI SDS was assessed by a stepwise regression analysis using SAS version 9.

## Results

### BHI and BHI SDS of BoneXpert

The mean BHI for the cohort was 4.73 ± 0.83 (2.36–9.7), while the mean BHI SDS was—0.79 ± 1.53 (- 6.4–2.9). BHI SDS readings for boys and girls did not significantly differ (boys (n = 189): -0.88 ± 1.5 (-5.58–2.88) girls (n = 119): -0.66 ± 1.59 (-6.4–2.19).

As expected, the BHI readings were positively correlated with patient age (r = 0.54; *P <* 0.0001), height (r = 0.57; *P* < 0.0001), weight (r = 0.52; *P <* 0.0001), BMI (r = 0.33; *P <* 0.0001) and testicular volume/breast development stage SDS (TVBR SDS, r = 0.24; *P* = 0.001) for the entire cohort. The BHI SDS was independent of age (*P =* 0.58), weight (*P =* 0.052), BMI (*P =* 0.11) and body height (*P* = 0.051), although it showed a weak positive correlation with testicular volume/breast development stage SDS (TVBR SDS, r = 0.18; *P =* 0.02). In a stepwise regression analysis, using two models containing either height SDS, weight SDS and pubertal status or BMI SDS and pubertal status, only height SDS could be shown to have a small effect on BHI-SDS (partial r^2^ = 0.015).

### Comparison of BHI (BoneXpert) with DXA readings

**Fig 1:**
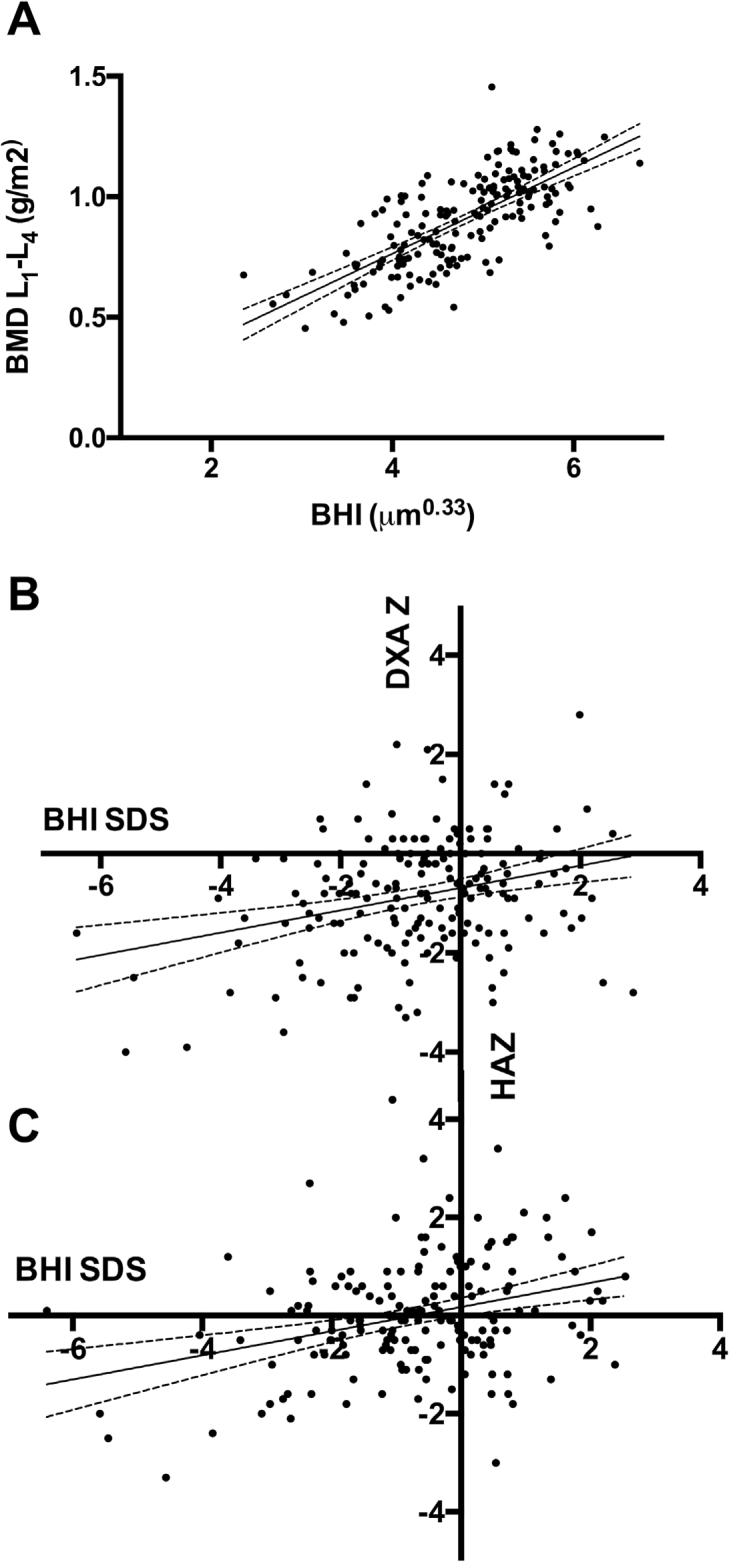
Positive association of BMD values obtained via BoneXpert and DXA scan. A) BMD readings of the lumbar spine (L1-L4) obtained by Lunar Prodigy (GE Healthcare) as a function of BHI obtained by BoneXpert (r = 0.73; P < 0.0001). B) Age-adjusted DXA Z-scores as a function of BHI SDS obtained via BoneXpert (r = 0.23; P = 0.02). C) DXA-Z scores, adjusted for patient height (HAZ) as a function of BHI SDS using BoneXpert (r = 0.27; P < 0.0001). For all figures, the predicted values based on bivariate regression analysis are indicated as solid line. The dashed lines represent the 95% confidence interval.

There was a significant and strong positive correlation between the BHI values of the left hand as assessed using BoneXpert and the areal BMD of vertebrae L1 –L4 as assessed via DXA (r = 0.73; *P <* 0.0001; Fig 1A). There was also a significant positive correlation between the BHI readouts and the BMD of the femoral neck as assessed via DXA (r = 0.58; *P <* 0.0001) for the entire group.

The age-adjusted DXA Z-scores of L1-L4 displayed a significant positive correlation with the BHI SDS (r = 0.23; *P =* 0.002, Fig 1B), as did the Z-scores for the femoral neck and the BHI SDS r = 0.23; P = 0.02. The height-adjusted DXA L1-L4 HAZ-scores and femoral neck HAZ scores also showed a significant positive correlation with the BHI SDS (r = 0.27; *P <* 0.0001 and r = 0.30; P = 0.002, respectively). Overall, HAZ-scores displayed a better correspondence with the BHI SDS than the Z-scores (Fig 1C).

In 12.5% of the analyzed pairs with a BoneXpert reading and a DXA result, the BHI SDS and the DXA Z-score differed > 2 SDS. After correction for height (using the HAZ score), the discordant results were only present in 4.1% of the pairs. The results of the DXA measurements, including Z-scores and HAZ-scores, are displayed in Table 2.

**Table 2:** Results of the DXA measurements.

	All	Boys	Girls
**BMD L1 –L4 (g/cm**^**2**^**)**	0.90 ± 0.19 (0.454–1.455) 178	0.86 ± 0.19 (0.454–1.261) 108	0.98 ± 0.18 (0.53–1.455) 70
**L1-L4 Z-score**	-0.83 ± 1.17 (-4.0–2.8) 182	-0.91 ± 1.16 (-4.0–2.1) 109	-0.7 ± 1.2 (-3.3–2.8) 73
**L1-L4 HAZ-score**	0.01 ± 1.17 (-3.3–4.4) 175	-0.18 ± 0.97 (-3.3–2.4) 105	0.3 ± 1.38 (-3–4.4) 70
**BMD femoral neck (g/ cm**^**2**^**)**	0.85 ± 0.17 (0.17–1.24) 102	0.85 ± 0.15 (0.59–1.18) 61	0.85± 0.20 (0.17–1.24) 41
**Femoral neck Z-score**	-0.69 ± 1.30 (-6.43–2.21) 102	- 0.59 ± 1.20 (-3.69–1.94) 61	-0.84 ± 1.43 (-6.43–2.21) 41
**Femoral neck HAZ-score**	-0.4 ± 1.33 (-7.6–1.8) 102	-0.31 ± 1.71 (-3.5–1.8) 61	-0.53 ± 1.55 (-7.6–1.8) 41

Mean ± SD and range (in parentheses) are displayed, followed by the number of patients for the following parameters: areal bone mineral density (BMD) for lumbar spine vertebrae (L1-L4), and femoral neck. Age corrected Z-scores (Z-score) and height adjusted score (HAZ score) are provided.

### Comparison of BHI readings (BoneXpert) with pQCT readings

**Fig 2:**
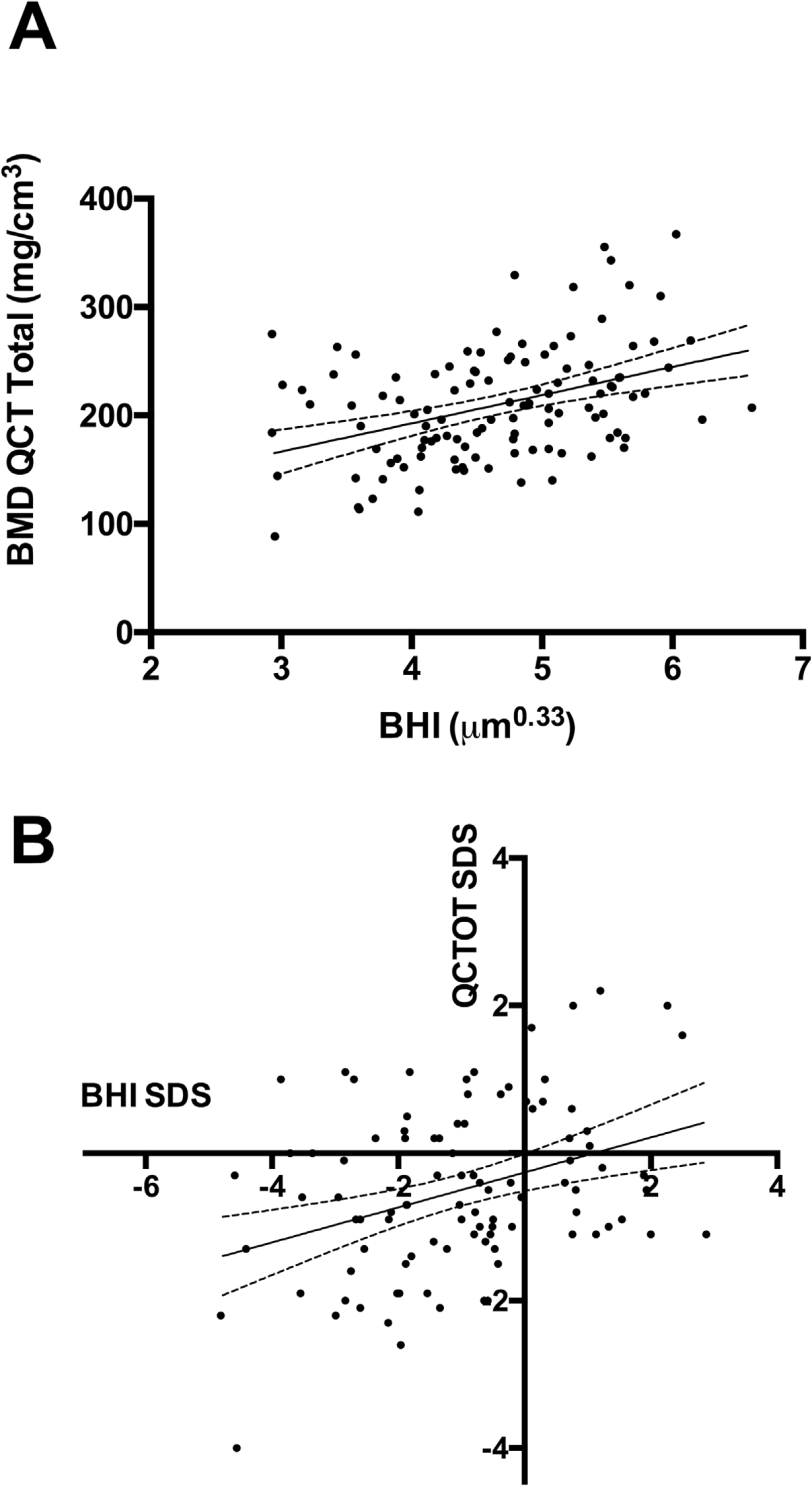
Positive association of BMD values obtained via BoneXpert and pQCT (XCT 900 Stratec®). A) BHI readings obtained using BoneXpert showed a positive correlation with BMD readings at the distal radius as measured via pQCT (XCT 900 Stratec®; r = 0.39; P < 0.0001). B) Accordingly, the SDS of the pQCT method (QCTOT SDS total, vertical axis) and the BoneXpert (BHI SDS, horizontal axis) were positively correlated (r = 0.3; P = 0.003). The predicted values based on bivariate regression analysis are indicated as a solid line. The dashed lines represent the 95% confidence interval.

The total BMD as assessed by pQCT showed a significant positive correlation with the BHI readings of BoneXpert (r = 0.39; *P <* 0.0001; Fig 2A), whereas trabecular BMD values less strongly correlated to BHI readings (r = 0.21; *P =* 0.02).

The age-adjusted scores for the pQCT readings also showed significant positive correlations with the BHI SDS values. The BHI-SDS values positively correlated with total pQCT-BMD SDS (r = 0.3; *P =* 0.003; Fig 2B) and less strongly with the trabecular BMD SDS (r = 0.22; *P =* 0.027) for the entire group.

The values of the pQCT readings and SD scores are displayed in Table 3. In 7.1% of the analyzed paired results of a BHI and a pQCT reading, the respective SDS values differed > 2 SDS.

**Table 3:** Results of the pQCT readings.

	All patients (n = 117)	Boys (n = 65)	Girls (n = 52)
**BMD total (mg/cm**^**3**^**)**	209.99 ± 52.54 (88.5–367) 117	203.62 ± 54.85 (88.5–343) 65	217.95 ± 48.85 (142.2–367) 52
**BMD total SDS**	-0.56 ± 1.12 (-4.0–2.2) 116	-0.63 ± 1.27 (-4.0–2.2) 64	-0.46 ± 0.92 (-2.1–2.0) 52
**Spongiosa (mg/cm**^**3**^**)**	132.95 ± 54.24 (36.7–325.7) 116	125.34 ± 51.36 (36.7–293.4) 64	142.32 ± 56.69 (38.0–325.7) 52
**Spongiosa SDS**	-1.55 ± 2.08 (-7.6–4.0) 116	-2.24 ± 2.12 (-7.6–2.9) 64	-0.69 ± 1.68 (-5.0–4.0) 52

Mean ± SD and range (in parentheses) are displayed, followed by the number of patients for the following parameters: volumetric bone mineral density (BMD total) of the distal left radius. Age-corrected values (BMD total SDS) and the respective results for spongiosa and spongiosa SDS are provided.

### Comparison of biochemical parameters of bone turnover and BHI reading

In a subgroup of patients (N = 114), biochemical parameters of bone turnover were available at the time the hand X-rays were taken. Analysis of these data revealed a vitamin D deficiency (serum 25 OH vitamin D < 20 ng/ml, as defined by the Institute of Medicine)[30] in 61.4% of the patients and a severe vitamin D deficiency (serum 25 OH vitamin D < 10 ng/ml[31]) in 23.9% of patients. Meanwhile, the 1,25 (OH)_2_ vitamin D serum levels were mostly normal. A secondary hyperparathyroidism had developed in 6.5% of the patients, and the total serum alkaline phosphatase (TSAP) and bone specific alkaline phosphatase (BAP) levels were elevated in 20.2% and 29.5% of patients, respectively. Other parameters of bone turnover were mostly within the age-appropriate norms. The biochemical characteristics of bone metabolism in this cohort are summarized in Table 4.

**Table 4:** Fraction and percentage of altered biochemical bone turnover markers (compared with the age-appropriate norm).

	All (n = 114)		Boys (n = 68)		Girls (n = 46)	
	Fraction	Percent	Fraction	Percent	Fraction	Percent
**25 OH VD <20**	70/114	61.4	41/68	60.3	29/46	63
**25 OH VD <10**	30/114	26.3	19/68	27.9	11/46	23.9
**PTH ↑**	12/110	10.9	7/67	10.4	5/43	11.6
**Ca:Crea ↓**	1/99	1	1/62	1.6	0/37	0
**TSAP ↑**	22/109	20.2	19/65	29.2	3/44	6.8
**BSAP↑**	33/112	29.5	27/68	25.2	6/44	13.6
**DPD/NTX ↑**	1/76	1.3	1/45	2.2	0/31	0

Fraction and percentage are presented for the following altered biochemical characteristics of bone metabolism: vitamin D deficiency (25-OH VD <20 ng/mL, including patients with severe vitamin D deficiency), severe vitamin D deficiency (25-OH VD <10 ng/mL), elevated parathyroid hormone (PTH), decreased calcium excretion in the urine (Ca:Crea < 0.03 mg/mg), elevated total serum alkaline phosphatase (TSAP), elevated bone-specific alkaline phosphatase (BAP) and elevated levels of N-terminal telopeptide (NTX) and urinary desoxypyridinoline (DPD).

There was a significant negative correlation between the BHI obtained using BoneXpert with the urinary deoxypyridinoline (DPD) (r = - 0.29; *P =* 0.026; n = 59), TSAP (r = - 0.2; *P =* 0.041; n = 105), BAP (r = - 0.26; *P =* 0.006; n = 108) and serum phosphorus levels (r = - 0.25; *P =* 0.01;, n = 106). Of these markers, only the serum phosphorus levels showed a significant correlation with the BHI-SDS values (r = 0.21; *P =* 0.027; n = 103).

Similar correlations were observed for the markers of bone turnover and the DXA areal BMD readings of L4; a significant negative correlation was found with DPD (r = - 0.42; *P* = 0.003; n = 46), TSAP (r = - 0.3; *P =* 0.004; n = 79), BAP (r = - 0.35; *P =* 0.001; n = 81) and the serum phosphorus levels (r = - 0.36; *P =* 0.001; n = 80). None of these parameters were associated with the age- or height-corrected DXA scores.

Due to the limited sample size, we were unable to perform a correlation analyses for the bone turnover markers and the pQCT results. Other biochemical parameters of bone turnover or bone health did not show a significant correlation with bone mineral density as assessed using the different techniques.

### Clinical characteristics of the patients

Clinical characteristics of the 337 patients, whose hand x-rays were reviewed, were assessed. The mean age at the time of x-ray was 13.58 ± 3.64 (3.64–21.14) years. The mean bone age, as assessed by the Greulich and Pyle method, was 12.75 ± 3.58 (2.42–19.0) years. The mean height SDS was -0.87 ± 1.18 (-4.06–2.73), the mean weight SDS was -0.30 ± 1.64 (-5.32–4.11) and the mean BMI SDS was 0.17 ± 1.58 (-5.65–4.62). Pubertal development SDS for pubic hair was -0.38 + 1.11 (-3.14–2.33) and for testicular volume (in boys) or breast development (in girls) was -0.6 ± 1.36 (-4.0–2.08).

The descriptive statistics are summarized in Table 1. The results for age, weight SDS, height SDS, BMI SDS, PH SDS, TVBR SDS and bone age, as assessed via the Greulich and Pyle method, did not differ significantly between boys and girls.

**Fig 3:**
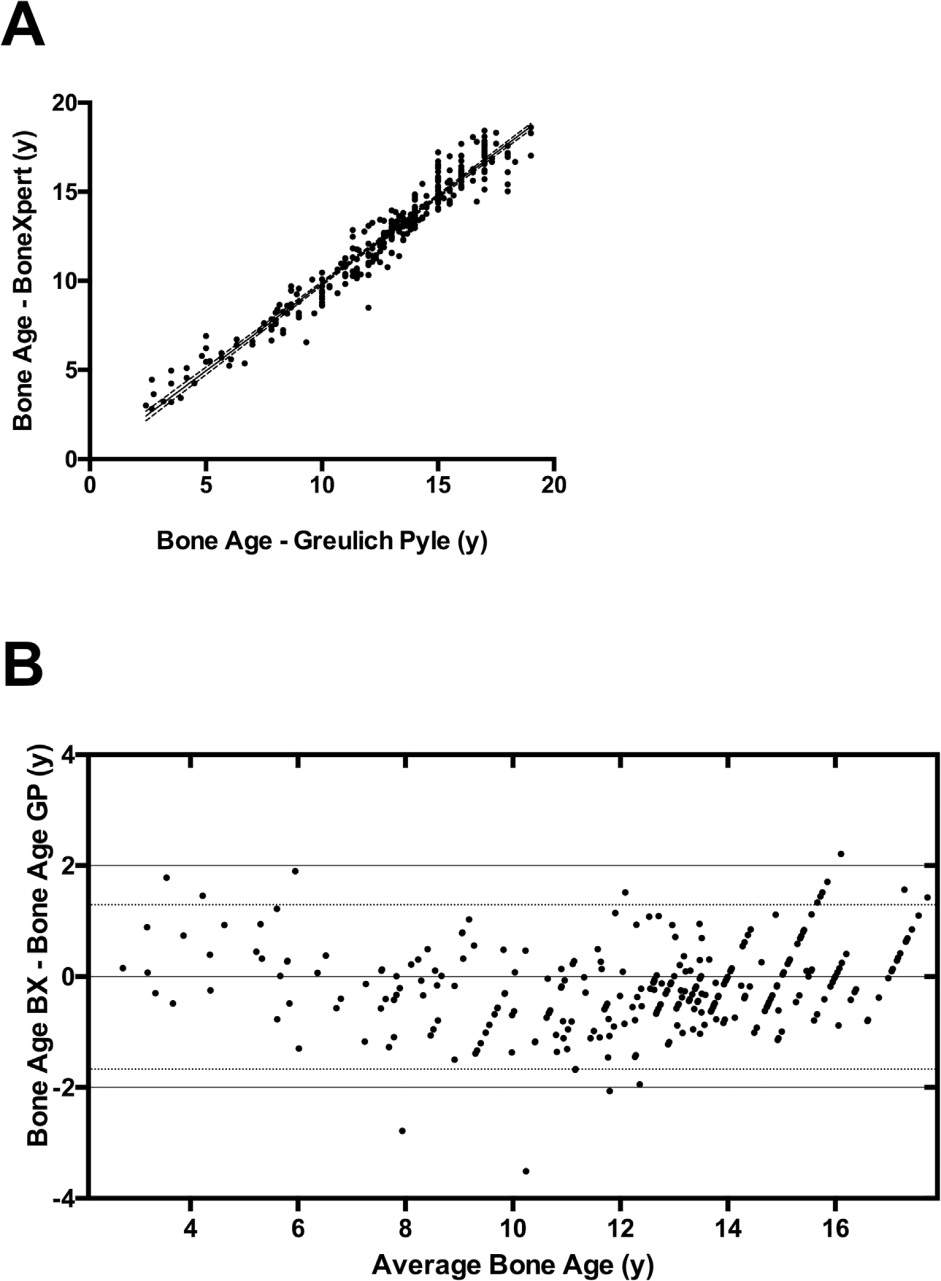
Comparison of manual and automated measurement of bone age. A) Bone age determined using BoneXpert as a function of manually measured bone age (Greulich and Pyle method). The predicted values, based on bivariate regression is indicated by the continuous line, the dashed lines represent the 95% confidence interval; r = 0.97; P < 0.0001). B) Bland Altman plot indicating the difference between automatic and manual rating for the licensed range of application of the BoneXpert software. The average of the two rating methods is shown along the horizontal axis, the difference is indicated along the vertical axis. The dotted lines represent the 95% limits of agreement (bias = -0.188; SD of Bias 0.76).

Skeletal age, as determined by the method of Greulich and Pyle, correlated well with the BHI results, as determined by the pediatric radiologist and BoneXpert (r = 0.97; *P* < 0.0001; Fig 3A and 3B).

## Discussion

Assessment of bone health in pediatric patients with chronic disease relies on the interpretation of clinical, biochemical and radiographic surrogate parameters by the treating physician[32–35]. One important piece of the puzzle is the assessment of BMD in children and adolescents[2,36,37]. The present study was undertaken to assess how the BHI[17] embedded in the program BoneXpert compares with pediatric bone mineral density readings assessed by two other methods, DXA and pQCT. We also aimed to determine whether the biochemical markers of calcium/bone metabolism corresponded to the BHI readings.

Our data indicate that BoneXpert provides BHI results are in concordance with the results of DXA or pQCT measurements for most patients. The BHI values showed the strongest positive correlation with areal BMD of L1-L4 as obtained via DXA scan. In children, the posterior-anterior lumbar spine and total body less head are the preferred sites to measure the areal BMD and to calculate the age-dependent Z-score. Currently no pediatric reference data for the femoral sites is being used. (for details see the position statement of the International Society for Clinical Densitometry (ISCD) from 2013)[2].

The BHI (BHI = T/(LW)^0.33^) is a further refinement of the concept of bone indices. The cortical thickness (T) can be interpreted as representing the areal bone density, while metacarpal index[7] and Exton-Smith´s[8] index represent the volumetric density. Thus, the BHI falls between areal (g/cm^2^) and volumetric density (g/cm^3^), as it is obtained by dividing T by smaller powers of length and width of the bone *(*g/cm^2.6666^).

Accordingly, a direct comparison of BHI, DXA and pQCT measurements is a comparison between three different methods. To account for the different methodologies, age-adjusted scores for the three methods (BHI SDS, DXA Z-score and pQCT-SDS) were compared. After calculation of the respective SDS values, the BHI SDS positively correlated with the DXA Z-score as well as the pQCT-SDS. However, the correlation coefficients were much stronger for the raw data, than for the standardized scores. This can, at least partly be attributed to the different reference populations which have been used for the generation of the standardized scores for each of the different techniques.

As bone mineralization in childhood and adolescence depends on age, gender, height, weight and pubertal development[2,38], the interpretation of bone mineral density readings requires automated or manual correction of these parameters. Regarding DXA results, the height dependency has been shown to be the limiting factor when interpreting the provided Z-scores[39]. Furthermore, different adjustments for the various DXA systems have been published[2,40–42].

Interestingly, in this study the BHI SDS showed a stronger and more significant correlation when compared with the height-corrected HAZ-score of the DXA measurements than with the age-corrected Z-score. This indicates the actual height of an individual has less influence on the BHI SDS results than on the DXA Z-score. BHI SDS may therefore present major advantages in the assessment of bone health in children.

Accordingly, the BHI SDS has been studied in patients with Klinefelter syndrome[43], growth hormone deficiency[44], Marfan syndrome [45] and juvenile idiopathic arthritis[46].

BHI and BHI SDS values also positively correlated with the total bone mineral density as assessed via pQCT. The correlation with the pQCT raw data was stronger than the correlation with the DXA raw data. This finding aligns with our hypotheses, as the results of BoneXpert and pQCT scans were both obtained at peripheral sites (metacarpals of the left hand versus distal (left) radius) and both methods analyze cortical thickness.

The trabecular structures are not well reflected by the BHI of BoneXpert, which thereby resulted in weak correlation coefficients for trabecular bone mass as assessed via pQCT and BHI. In this aspect, pQCT analysis provides more in depth data concerning bone mineralization during childhood and adolescence.

Hand X-rays of patients with bone mineralization defects, especially defects affecting the cortical bone, remain unsuitable for analysis using BoneXpert. This problem affected < 3% of the images that were sent for automated calculation of the BHI in this study. Thus, the ratio of images that were rejected by the BoneXpert software was slightly higher than previously described[19] and lower than that reported using Pronosco/Sectra X-posure System, as published by van Rijn et al in 2004[47]. Amongst the children with rejected images were children with underlying skeletal disorders, including a patient with juvenile Paget Syndrome[48] involving ossification defects. The slightly higher rejection rate observed in this study might be caused by the subgroup of children with skeletal dysplasia or calcium metabolism disorders.

While the results for BHI and DXA or pQCT scans were positively correlated, the readings for some patients differed greatly. We further investigated individual cases when SDS readings differed by > 4 SDS between BHI-SDS and DXA Z-score or pQCT-SDS readings.

In two patients, who were labeled as osteopenic with an age-corrected Z-score < -2 in the DXA analysis, the BHI SDS were +2.4 and +2.9, respectively. A review of the patient data revealed that both patients were of small build (height SDS -2.08 and -1.75; weight SDS -2.26 and -2.73, respectively). To assess whether the short stature was the underlying reason for the very low DXA-Z scores, we assessed the height-adjusted HAZ-scores, which were indeed within the normal range (-0.1 and -0.5, respectively). However, even after this correction for height, the findings between DXA and BoneXpert still differed > 2 SDS. The underlying reason for the discrepancy might be due to the delayed bone ages (> 3 years and > 2.5 years, respectively) of the patients.

Overall, divergent results (>2 SDS) were found in approximately 12% of the comparisons of BHI SDS with DXA Z-score, 4% with HAZ score and 7% with pQCT SDS. The underlying reason for the divergent results and rejected X-rays warrants further evaluation.

An association of the BMD as assessed via DXA and 25-OH vitamin D level measurement has been previously demonstrated in larger pediatric cohorts [49]. However, in this study, parameters of bone turnover or calcium metabolism were not found to be associated with the BHI SDS or the DXA Z-scores. This finding is not surprising, as bone mineral density and cortical thickness are expected to be stable parameters, which are not dependent on temporary changes in bone turnover.

### Limitations of the study

The present study has some limitations. It is a small retrospective study of available DXA and pQCT results obtained in children in whom also an X-ray of the left hand was obtained to assess the bone age. Since these investigations (X-ray, DXA and pQCT) were often done on separate clinic visits a time interval of 8 months maximum between the X-ray of the left hand and the DXA or the pQCT assessment was defined. This resulted in a mean time difference between the X-ray of the left hand and the bone-density scans of 0.2 months for the DXA group and 0.3 months for the pQCT group. However, some individuals had longer time-intervals, of up to 8 months. This study is not designed to investigate the clinical use of the BoneXpert to predict fracture risk.

## Conclusions

The present study demonstrates that the BHI and BHI-SDS of the BoneXpert software largely correlate with the DXA or pQCT readings. Thus, the BHI appears to be a useful tool to obtain additional information concerning bone health in children with suspected bone disease, potentially sparing additional irradiation. Furthermore, the BHI may represent a valuable screening tool to monitor chronically ill children and adolescents. Pathological results on the routinely ordered hand X-rays could help to identify impaired bone health and prompt further diagnostic workup.

## Abbreviations

25-OH VD: Serum levels of 25-OH vitamin D
BMD: Bone mineral density
BMI: Body mass index
BSAP: Bone-specific alkaline phosphatase
CACRERAT: Calcium to creatinine ratio in urine
DXA: Dual-energy X-ray absorptiometry
HAZ: Height-adjusted Z-score
pQCT: Peripheral quantitative computed tomography
PTH: Parathyroid hormone
SDS: Standard deviation score
TSAP: Total serum alkaline phosphatase
Z-score: Age-corrected standard deviation score
